# Navigating recurrent *Clostridioides difficile* infection without Bezlotoxumab: potential challenges ahead

**DOI:** 10.1017/ash.2025.10144

**Published:** 2025-12-09

**Authors:** Radhika Arya, Alex N Zimmet, Marisa Holubar, William Alegria

**Affiliations:** 1 Division of Infectious Diseases and Geographic Medicine, Department of Medicine, Stanford University School of Medicine, Stanford, CA, USA; 2 Department of Quality, Safety & Health Equity, Stanford University School of Medicine, Stanford, CA, USA

*Clostridioides difficile* infection (CDI) remains a leading cause of healthcare-associated diarrhea, with 20%–35% of patients developing recurrent infection.^1^ Over the past decade, diagnostic and antimicrobial stewardship interventions have been credited with reducing rates of CDI.^1^ Nonetheless, recurrent *Clostridioides difficile* infection (rCDI) remains a challenge with rising cases annually contributing to significant morbidity and mortality.

Bezlotoxumab, a human monoclonal antibody targeting *Clostridioides difficile* toxin B, helps prevent toxin mediated damage of the colonic epithelium. It was first approved by the Food and Drug Administration (FDA) in 2016 as an adjunctive therapy to reduce CDI recurrence in adults. In licensing studies, bezlotoxumab was associated with rCDI rates of 16% compared to 26% with placebo at 12 weeks 95% CI, −15.5 to −4.3; *P* < 0.001) when given in addition to standard of care antibiotics, most commonly oral vancomycin.^2^ The 2021 national guidelines endorsed its use as a cointervention with standard of care antibiotics in patients with first or subsequent recurrence of CDI.^3^ These guidelines acknowledged the logistic barriers complicating clinical use but noted a high value on potential clinical benefits, especially in patients with risk factors for rCDI such as age ≥ 65 years, immunosuppression, and severe CDI on presentation.

Despite this, bezlotoxumab was withdrawn from the market by its sole supplier on January 31, 2025. The withdrawal of this drug raises concerns regarding the future management and prevention of rCDI. There are two other practices currently in place for prevention of rCDI—oral vancomycin prophylaxis (OVP) and fecal microbiota-based therapies (FMT), each with distinct advantages and limitations that guide their use in clinical practice.

OVP is commonly used as a preventive strategy for rCDI.^4^ Evidence supporting OVP use is primarily limited to retrospective studies that show modest benefit.^5,6^ These studies are often constrained by small sample sizes and significant variability in dosing regimens and duration of therapy, making it difficult to derive standardized recommendations for clinical use or to establish optimal prophylactic protocols. Additionally, most studies evaluating OVP have limited follow-up periods, potentially missing subsequent rCDI that may occur shortly after discontinuation of OVP due to worsened gut dysbiosis.^7^ A systematic review with considerable heterogeneity that included 11 retrospective studies and 1 randomized controlled trial demonstrated that OVP was associated with lower risk of rCDI compared to no prophylaxis (6.4% vs 19.2%, *p* < 0.001), with the greatest benefit observed among patients with prior CDI within 90 days of the index admission.^8^ Despite these findings, the routine use of OVP has not yet been established as standard of care, given the limitations of existing evidence.^9^ The prolonged or repeated use of oral vancomycin may further disrupt the gut microbiota, including the potential for colonization with vancomycin-resistant Enterococci (VRE). However, data on this risk are inconsistent.^10–14^ Other potential downsides, such as cumulative drug exposure, increased healthcare costs, and the risk of fostering antibiotic dependency for CDI prevention remain unknown, underscoring the need for more robust prospective studies to guide clinical practice.

FMT, which include conventional FMT and live biotherapeutics (fecal microbiota live-jslm (REBYOTA) and fecal microbiota spores live-brpk (VOWST)) have emerged as novel agents for management of rCDI. From the landmark randomized controlled trial in 2013 demonstrating the benefit of duodenal infusion of donor feces in rCDI,^15^ to the introduction of commercial FMT products, the world of FMT is rapidly evolving. The American Gastroenterological Association (AGA) recommends considering the use of FMT in immunocompetent patients with two or more episodes of rCDI, or selectively in those at high risk for rCDI.^16^ Conventional FMT is available through nonprofit stool banks within select academic centers, and FDA-approved live biotherapeutic FMT are commercially accessible. Despite this, widespread adoption remains constrained by practical barriers including high costs and insurance coverage, which previously affected the uptake of Bezlotoxumab.(Table 1) Recent studies suggest that live biotherapeutic FMT may still represent a cost-effective intervention by reducing recurrence rates, hospital readmissions, and associated healthcare utilization costs.^17^ Additionally, the current data on efficacy of live biotherapeutic FMT is subject to significant bias based on funding sources. Further real-world studies are essential to accurately assess the clinical and economic impact of integrating commercial FMT products into mainstream practice.

**Table 1.** Cost and specifics of commercial products for prevention of rCDI

Drug/Formulation	Cost*	Specifics of administration	Data supporting use	Considerations
Fecal microbiota live-jslm (REBYOTA)	$9,135 per unit	Administer 24 to 72 hours after the last dose of antibiotics for CDI Single dose administered per rectum	Randomized controlled trial (PUNCH CD3 trial)^27^	Rectal administration by healthcare provider Bowel prep required Not recommended for use in immunocompromised individuals No antibiotic use up to 8 weeks from administration
Fecal microbiota spores live-brpk (VOWST)	$19,106.50 per course of therapy (12 capsules)	Administer 2 to 4 days after last dose of antibiotics for CDI 4 capsules PO once daily for 3 days	Randomized controlled trial (ECOSPOR III and ECOSPOR IV)^28,29^	Self-administration No bowel prep required Not recommended in immunocompromised individuals No concomitant antibiotic use
Bezlotoxumab	∼$4000 (40 ml)	Single dose of 10 mg/kg IV during antibacterial drug treatment for CDI	Randomized controlled trial (MODIFY I and II trial)^2^	Parenteral administration by healthcare provider No bowel prep required Recommended in immunocompromised individuals Concomitant antibiotic use allowed

* Includes drug WAC (Wholesale acquisition cost).^23^ Nursing time/administration and facility fee must also be considered.

The AGA guidelines primarily recommend live biotherapeutic FMT for non-severe, non-fulminant rCDI in the outpatient setting, leaving limited therapeutic options for use in hospitalized patients. In the inpatient setting, conventional FMT has demonstrated benefit in a select subset of patients with severe or fulminant CDI, with retrospective studies reporting clinical cure rates of 61.3% (95% CI, 43.2–78.0).^18^ But conventional FMT use remains challenging in hospitalized patients who require frequent or prolonged antibiotic courses, as ongoing antibiotic exposure may compromise the efficacy of microbiota-based therapies.^16^

It is unclear if FMT or OVP is as effective as bezlotoxumab in preventing rCDI given there are no comparative trials. A systematic review and Bayesian network meta-analysis of seven randomized controlled trials with 3,043 patients found no significant difference in rCDI resolution between conventional FMT and bezlotoxumab [OR 1.53 (95% credible interval 0.39–5.16) for FMT and 2.86 (95% credible interval 1.29–6.57) for bezlotoxumab], although patients receiving FMT had higher rates of diarrhea.^19^ Additionally, a Spanish retrospective study of 100 CDI episodes (51 with bezlotoxumab and 49 with FMT with lyophilized oral capsules) showed similar effectiveness in preventing rCDI, with recurrence rates of 9.8% for bezlotoxumab compared to 18.4% for FMT (P = 0.31).^20^ These studies are limited in their retrospective approach making it challenging to assess their comparative efficacy.

There is a lack of randomized trials directly comparing OVP with various FMT options, making it difficult to determine the relative efficacy of these preventive strategies. In a randomized controlled trial of 30 adults with rCDI, there was no significant difference in CDI recurrence within 120 days between patients treated with 14 days of oral vancomycin followed by a single FMT via enema and those who received a standard 6-week vancomycin taper.^21^ Additionally, no randomized studies have compared different FMT products to one another, further limiting guidance for selecting among available FMT options.

The loss of bezlotoxumab uniquely impacts management of rCDI in immunocompromised patients, a population with especially high rates of recurrence.^22^ The safety of FMT products in the setting of varying degrees of immunocompromise is not well known and thus their use in such patients is generally avoided. Additionally, these populations have high rates of antibiotic exposure and often may be reinitiated on antibiotics soon after completing CDI treatment. FMT products may be inactivated by subsequent antibiotics, leading manufacturers to recommend a period of time avoiding their use after administration (up to 8 weeks in the case of REBYOTA). This may not be feasible in highly comorbid immunocompromised hosts and further precludes use of novel FMT products in these patients. Finally, concerns about OVP contributing to further dysbiosis are exacerbated in immunocompromised oncology populations, in whom dysbiosis has already been linked to worsened cancer-related outcomes.^23,24^ Bezlotoxumab was previously a viable option to minimize recurrence risk in the face of these concerns—with its removal from the market, clinicians caring for these patients, in whom rCDI is especially common and impactful, are left with even more limited options to prevent recurrence.

The lack of data and significant logistic barriers to accessing available options leaves clinicians with limited options for rCDI. While judicious antimicrobial use remains essential, additional efforts are needed. Clinicians can help drive system-level change by advocating for fidaxomicin and FMT to be financially accessible without cost or insurance barriers, and by partnering with patient advocacy organizations to advance policy and awareness efforts.^25^ Additional studies evaluating use of FMT in the immunocompromised populations are needed, as well as exploring the role of new therapeutics and primary prevention through vaccination for the control of CDI globally.^26^
